# ‘Every medicine is part poison’: a qualitative inquiry into the perceptions and experiences of choosing contraceptive methods of migrant Chinese women living in Australia

**DOI:** 10.1186/s12905-021-01226-3

**Published:** 2021-03-08

**Authors:** Hankiz Dolan, Mu Li, Deborah Bateson, Rachel Thompson, Chun Wah Michael Tam, Carissa Bonner, Lyndal Trevena

**Affiliations:** 1 School of Public Health, The University of Sydney, Room 125, Edward Ford Building, Sydney, NSW 2006 Australia; 2 Ask, Share, Know: Rapid Evidence for General Practice Decision (ASK-GP), Centre for Research Excellence, The University of Sydney, Sydney, NSW 2006 Australia; 3 Family Planning NSW, Sydney, NSW 2131 Australia; 4 Discipline of Obstetrics, Gynaecology and Neonatology, The University of Sydney, Sydney, NSW 2006 Australia; 5 Primary and Integrated Care Unit, South Western Sydney Local Health District, Liverpool, NSW 2170 Australia; 6 School of Population Health, Faculty of Medicine, UNSW, Sydney, NSW 2052 Australia

**Keywords:** Contraception, Chinese migrant women, Decision-making, Critical realism

## Abstract

**Background:**

In Australia, ethnic Chinese people are one of the largest, youngest and fastest growing overseas-born groups. Yet, little is known about their perceptions of contraceptive methods and their experiences with choosing one. Decisions about contraceptive methods are preference sensitive. Understanding the influencing factors of Chinese migrant women’s contraceptive method choice and practices will help cater to their decision-making needs in a culturally sensitive and responsive way.

**Methods:**

A qualitative study design underpinned by critical realism approach was used to explore Chinese migrant women’s perceptions and experiences of choosing contraceptive methods. Semi-structured interviews were conducted with 22 women who self-identified as being ethnically Chinese and had been living in Australia for no more than 10 years. The interview guide was adapted from the Ottawa Decision Support Framework. Majority of the interviews were conducted in Mandarin Chinese. Transcribed data was analysed using thematic analysis method.

**Results:**

Four major themes were identified, including: ‘every medicine is part poison: hormonal contraceptives cause harm to the body’; ‘intrauterine device, a device used in the past for married women’; ‘it takes two (or one) to decide, depending on the relationship dynamics and contraception preferences’; and ‘it is not necessary to seek medical advice in choosing contraceptive methods’.

**Conclusions:**

Our findings suggest that Chinese migrant women’s perceptions and experiences of choosing contraceptive methods are influenced by complex personal, cultural, societal and inter-relational factors. Chinese migrant women were cautious of using hormonal methods due to fears of side-effects, including reduced or absent menstrual bleeding. Women were also reluctant to consider intrauterine devices as options due to associating them with past experiences of other women and themselves and also fears of potential complications. There was a reluctant attitude towards seeking medical advice regarding contraception due to beliefs that needing to use contraception is not an illness requiring treatment. Such findings are likely to be useful in increasing healthcare professionals’ and policy makers’ understanding of Chinese migrant women’s contraceptive method preferences, beliefs and behaviours. They also help to develop culturally and linguistically sensitive strategies, which goes beyond the provision of contraceptive counselling, in assisting Chinese migrant women’s decision-making needs.

**Supplementary Information:**

The online version contains supplementary material available at 10.1186/s12905-021-01226-3.

## Background

Australia is a multicultural country. Approximately one in four people who reside in Australia are born overseas[1]. In 2016, 5.6% of Australia’s total population identified themselves as having Chinese ancestry, while 2.2% identified as being born in China [1, 2]. Among the Australian population who were born in China, 62.3% held foreign nationality [3]. University students, including international students, accounted for 22% of the total China-born population [4]. The median age for people who were born in China was 33 in 2016 [3], making them one of the youngest, largest and fastest growing overseas-born groups in Australia.

Contraception is a key component of sexual and reproductive health [5]. The fundamental purpose of contraception is to prevent unintended pregnancies and increase people’s control over whether or not, and when to have children and how many to have [6]. In Australia, there are many types of contraceptive methods, including intrauterine devices (IUDs), implant, oral contraceptive pills, vaginal ring, condoms, diaphragm and male and female sterilisation, are available for people to choose from [7]. Among those methods, contraceptive pills are the most commonly used by women in Australia, followed by condoms and sterilisation [8]. Compared to Australian-born women, women from non-English-speaking backgrounds who were born overseas are less likely to report use of any contraceptive method [9]. They may face barriers in accessing evidence-based, culturally and linguistically sensitive health information, and are more likely to rely on family, friends or media for contraceptive information which may not be based on accurate medical evidence [10–12]. Apart from cultural and language barriers, misconceptions or concerns about contraception such as weight gain, bleeding, cancer risk and worries about side-effects were also reported by migrant and refugee women [12].

It has also been reported that international students, including those from China, have low sexual health literacy and limited knowledge about contraceptive methods [13–15]. They are reported to be reluctant to speak to healthcare providers about sexual and reproductive health matters due to fears of embarrassment and being unaware of healthcare providers’ potential role in providing sexual and reproductive health counselling and related services [13–15]. Little is known about Chinese migrant women’s, including Chinese international students’, perceptions and awareness of contraceptive methods and factors that influence their choice of contraceptives. Decisions about contraceptive methods are preference-sensitive [16]. Understanding the influencing factors of migrant Chinese women’s contraceptive method choice and practices will help cater to their decision-making needs in a culturally sensitive and responsive way. Therefore, we set out to explore Chinese women’s knowledge, perceptions and views of contraceptive methods, and their experiences with choosing one in Australia using a qualitative critical realist approach.

## Method

### Research team and reflexivity

The research team comprised of members with a wide range of expertise in areas such as general practice, sexual and reproductive health, maternal and child health, psychology and behaviour change. The study was conducted as part of the lead author’s (HD) doctorate project. HD is a female and is a medical graduate. She is fluent in English and Mandarin Chinese. She and all other team members had training in qualitative research prior to conducting the study. None of the team members had any personal or professional relationships or contact with the participants prior to the recruitment.

### Study design

This qualitative research was informed by the critical realism theoretical approach. Critical realism is based on the ontological assumption that reality consists of three layers: ‘empirical’ reality which is observed, experienced or perceived by humans; ‘actual’ reality of events which occur regardless of human knowledge; and ‘real’ reality of causal mechanisms such as social and cultural structures which shape the manifestation of empirical reality [17]. In the context of contraceptive method choice, the ‘empirical’ level concerns with beliefs, perceptions and experiences of women with the specific contraceptive methods. At the ‘actual’ level is the attributes, risks and benefits of contraceptive methods, which may or may not be known to the women. The ‘real’ reality level is the structural generative mechanisms and factors that cause women to come to perceive or experience contraceptive methods in certain ways. The critical realist approach goes beyond describing social phenomena and seeks to elaborate on fundamental underlying causes of such observed, experienced and perceived empirical reality [18]. We took a critical realism approach because we believed that women’s perceptions of contraceptive methods and their experiences of choosing one are manifestations of the interplay of social, cultural and individual contexts or mechanisms.

Given the potentially sensitive and personal nature of this topic, we chose semi-structured individual interviews for data collection. Semi-structured interviews are usually guided by pre-defined questions [19]. However, they also have the advantage of allowing the researcher to tailor the questions to each participant in order to ensure the natural flow of the conversation [19].

### Recruitment

We recruited women who self-identified as ethnically Chinese, aged between 18 and 45 years and had been living in Australia for no more than 10 years. We used a purposive sampling method to cover a diverse range of participant characteristics, including age (e.g., 18–30 years versus 30–44 years), Australian residency status (citizen, permanent resident versus temporary residents) and relationship status (married, living with a partner versus not-married, not-living with a partner). We utilised several recruitment methods. First, we placed Chinese and English versions of flyers and posters at our recruitment sites, including a university campus, university health clinic, family planning clinics and a Chinese community service centre. Second, we posted similar information on one of the largest Chinese Australian social networking sites, Oursteps. Third, HD approached potential participants directly in the waiting room of one of the non-profit family planning clinics in Sydney. Given the popularity of WeChat, a social media and messaging app, among people from Chinese background living in Australia, we registered a WeChat account on a study-specific mobile phone device and specified our WeChat details and QR code on our recruitment materials. When women contacted the research team expressing their interest in the study, we sent them plain-language ‘participant information statement’ and consent form in either Chinese or English language, depending on their preferences. Interviews were scheduled at a time convenient to the participants.

### Data collection

Interviews were conducted between July 2018 and January 2019. HD conducted all interviews with the participants at mutually agreed safe and private venues in metropolitan Sydney. Interviews were completed via telephone if preferred. Participants were given a choice to be interviewed either in Mandarin Chinese or English. Each interview was divided into two parts. In the first part, we we conducted the interview using a topic guide that was primarily adapted from the Ottawa Decision Support Framework [20], which included questions relating to contraception attitude, knowledge, perceptions, experiences, decisional conflict and support needs. The interview topic guide is provided as Additional file 1. We modified interview questions iteratively throughout the data collection. In the second part, women were shown a 8-page English and Chinese-translated contraceptive methods decision aid and probed to provide feedback. Only the data from the first part was analysed and reported for this paper. Participants each received a 30AUD shopping voucher for their participation.

All interviews except one were audio-recorded with the consent from the participants. One participant did not consent to be audio-recorded, and detailed field notes were collected instead. HD transcribed the recordings verbatim, and this process formed the early stages of the data analysis. We chose to transcribe the interview audio-recordings in the original language (i.e., either Chinese or English) to avoid loss of meaning due to translation [21]. Only one transcript was entirely in English. HD translated two additional Chinese transcripts into English for the view of other non-Chinese speaking team members. HD also translated the initial thematic table, which organises participant quotes under each potential candidate themes, for team discussion. Translation of key terms and concepts were discussed with the second author (ML) who is a native Chinese speaker.

### Data analysis

We followed the thematic analysis method [22] within a critical realism approach to analyse the data. As the first step of the analysis, HD transcribed and read all the transcripts while other team members read selected transcripts to familiarise themselves with the data. HD coded all transcripts using NVivo 12 [23] primarily in English. Some key codes were accompanied by source Chinese words and phrases. Other team members coded selected transcripts. Our initial codes were mainly driven by raw data inductively. HD wrote memos after coding each transcript to record data impression and analytical thinking. We held regular team meetings where we employed a peer debriefing strategy to review the data and analysis process, discuss the differential interpretation of the data and appraise the conceptualisation of research findings [24, 25]. We collapsed, clustered and organised the initial codes in a recursive manner to identify recurring patterns and candidate themes. We used thematic table and diagram to identify themes that were internally homogenous and externally heterogeneous. During theme development and preparation of final analysis report, we engaged with existing literature and theories (abduction)[26] and/or contextual and historical factors to move beyond the surface descriptions to seek explanations for key concepts, phenomena and causal mechanisms reflected throughout each theme (retroduction)[26]. As with critical realist epistemology, we took the stance that our explorations of such mechanisms are our attempts to approximate the ‘truth’ rather than to determine the ‘truth’ [26].

### Trustworthiness

We established trustworthiness and rigor during data analysis by: engaging with the data for a prolonged period of time; keeping reflective memos during data collection and analysis; frequent debriefing with team members; using thematic tables and diagrams to establish analytical reasoning; and providing thick description of the study methodology and the data.

### Ethical consideraion

We obtained ethics approval from the University of Sydney human research ethics committee [ref: 2018/159] and Family Planning New South Wales Project Ethical Risk Team [ref: PERT 25]. All participants read the participant information statement and consented to participate. Participation was voluntary and participants were informed that they can terminate the interviews anytime if they did not wish to continue.

## Results

**Fig. 1:**
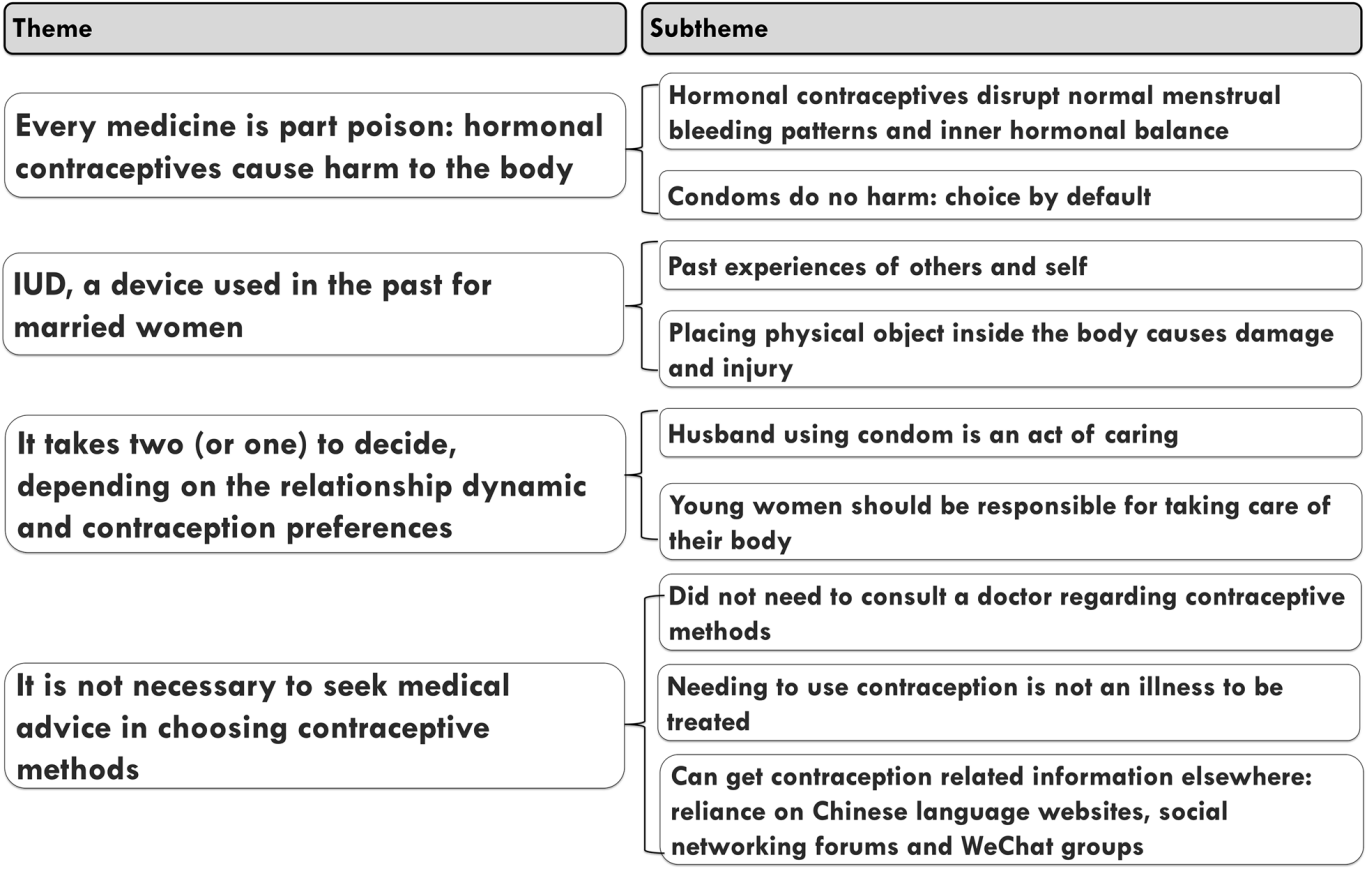
Overview of themes and subthemes

A total of 22 women who were living in Sydney metropolitan area took part in the interviews (Table 1). We conducted 16 face-to-face interviews and six phone interviews. Interviews lasted between 25 and 75 min. We identified four overarching themes, each with two to three subthemes (Fig. 1).

**Table 1:** Participant characteristics

	Number
Age group	
18–30	13
30–45	9
Australian residency status	
Australian citizen or permanent resident	9
Not an Australian citizen or permanent resident	13
Relationship status	
Married	10
Not married/not living with a partner	12
Place of birth	
Mainland China	20
Hong Kong, China	2
Employment status	
Employed	9
Student	10
Un-employed	3
Number of children	
None	15
1–3	7

### Theme one: ‘Every medicine is part poison’: hormonal contraceptives cause harm to the body

The Chinese proverb ‘every medicine is part poison’ (是药三分毒 Shì yào sān fēn dú) was repeatedly referred to by women expressing their beliefs and attitudes towards hormonal contraceptives, including oral contraceptive pills, hormonal IUDs, and progestogen-only implant. Most women were cautious of hormonal contraceptives because they believed that such medicines would disrupt their ‘inner balance’ and cause harm to their body. Such perceived harms included reduced or irregular menstrual bleeding, weight gain, acne and mood change. For many of our participants, it was implied that the benefits of avoiding irregular menstrual bleeding and internal imbalance outweighed the benefits of using hormonal contraceptives, (i.e., to prevent pregnancy), especially when there were other ‘safer’ options available.

I always feel that every medicine is part poison, that is why I feel they [contraceptive pills] might, if you take them for long term, might have some impact on some body organs… I believe that and I just have that sort of fear, I just oppose taking pills

(Participant 4, married, aged 30–45, one child)

### Sub-theme 1a: Hormonal contraceptives disrupt normal menstrual bleeding patterns and inner hormonal balance

One of the most frequently mentioned negative impacts of the hormonal contraceptives was reduced or irregular menstrual bleeding. Some women perceived this to be just one amongst several disadvantages of the hormonal contraceptives. Others had deliberately discontinued hormonal methods after a short period time of ‘giving it a try’ and experiencing this particular negative impact. When probed about the reasons for avoiding side-effects related to reduced or absent menstrual bleeding, women cited several personal beliefs. For some women, having monthly periods was seen as a sign of female fertility and womanhood. Reduced or absent menstrual bleeding were also perceived as signals of menopause, which would accelerate the female aging process. As one woman quoted:

As a Chinese woman, having monthly menstrual bleeding cycle is a sign of your fertility. If you suddenly don’t have any periods, wouldn’t it feel like you are having menopause…if I tell my mother that I stopped having periods after taking the pill, she would think that I am having menopause and I can’t have any child in the future…she would have that sort of concerns and she would say, you have lost an important feature of womanhood

(Participant 19, married, aged 18–30, no children)

Others saw that having reduced, absent or irregular bleeding was a sign of underlying health problems. For example, one woman noted that she had an irregular menstrual cycle even without taking the contraceptive pills and believed the irregularity was related to her ‘body constitution’. Another woman was strongly against taking oral contraceptive pills because she felt they ‘harm the female body to a great degree’. However, she had favourable attitudes towards the use of Chinese medicine in regulating menstruation. As she described:

[having period] is supposed to be a normal physiological thing…if suddenly there is no period, it feels very scary, and you would want to find out the reasons why, first you might go and do a pregnancy test to see if you are pregnant or not, if not pregnant, you would look for other reasons, just like my friend, if her period does not come or the amount is very little, she would buy medicines to help regulate her periods, we Chinese people all like taking Chinese medicines and things like that’

(Participant 10, not married, aged 18–30, no children)

### Sub-theme 1b: Condoms do no harm: choice by default

The use of male condoms was favoured by many of the participants, regardless of their marital status or whether or not they had given birth before. It was often described as a method which was convenient, easy to use, clean and efficient. Most importantly, for many participants, the male condom is a barrier method that stayed outside one’s body and therefore it does not inflict ‘harm’ to the female body like the hormonal contraceptives do.

I don’t like taking pills and things like that, because I always feel that things [medicine] you ingest would always cause some sort of harm to you, therefore, I am not very willing to take pills, I usually just use condoms…because you ingest things [as in take pills], I mean, if you could replace a method which requires you to ingest, then I think condoms are better, at least they stay outside

(Participant 14, not married, aged 18–30, no children)

Apart from the use of condoms, use of the ‘safe period’ method was also frequently mentioned by the participants. The common term they used to describe the safe period method was ‘before seven after eight’*,* which refers to a variation of calender method where 7 days before and 8 days after the first day of menstruation are considered safe to have unprotected sex. As one participant described:

My period is usually ok, relatively regular, but I would always count from the day when my period arrived, because my period is for 6 days, I count from that [first] day, then I add another 8 days and then I start using condoms

(Participant 8, married, aged 30–45, one child)

A few participants mentioned how they use a combination of condoms, safe period and withdrawal methods. Overall, the majority of participants preferred the use of condoms, sometimes in combination with ‘natural’ methods such as withdrawal or safe period method. For them, such combination not only delivered the benefit of avoiding pregnancy, but also safeguarded against ‘sufferings’ caused by using hormonal methods.

In China, withdrawal method is relatively popular, in fact, I know that the withdrawal method, it is not that effective, but, it may not sound very scientific, but for me, I rarely have periods, so, the chance of me falling pregnant is not big, and I only have one sex partner, two of us, then, first, we choose a safe time, second, he withdraws, in fact, with using condoms, it is 1:1 ratio

(Participant 2, not married, aged 18–30, no children)

### Theme two: The IUD, a device used in the past for married women

This theme mainly captured women’s perceptions around non-hormonal IUDs. It is important to note that many participants did not differentiate between the two types IUDs (hormonal versus non-hormonal) and they used the term ‘*ring*’ to describe all intrauterine contraceptive methods. IUDs are commonly known as ‘contraceptive rings’ in China because the earliest intrauterine devices that were widely used during the initial periods of the nationwide one-child policy were largely ‘home-made’ stainless-steel rings (SSR) [27]. SSRs were designed not to be removed after a first delivery [27]. It was in 1993 that China ceased manufacturing SSRs [27, 28]. To ensure the term ‘ring’ was not misinterpreted as referring to the hormonal vaginal ring currently available in Australia, we replaced the literal translation of ‘ring’ with ‘IUD’.

### Sub-theme 2a: Past experiences of others and self

When speaking of their knowledge and perceptions of IUDs, many women referred to the experiences of their mothers, female relatives or acquaintances of having IUDs. Some participants expressed their impression of the IUD being a contraceptive method of the past when married women were mandated to have them to prevent having further children. They expressed their impression and perceptions of the IUD being a device that causes pain and harm to the female body. In the accounts of those women, there was a general sense of rejection of IUDs as a sensible contraceptive option for them. It was implied that IUDs do not apply to their current situations where they are not constrained by family planning policies or they are not married or had children yet.

Honestly, I don’t know how this thing work, I feel like in the old days, in China, if they did not allow you to have children anymore, some people in the villages would have this thing, but it must be quite painful. If you think about it, you put a thing inside [your body], just stretching it in causes pain, just thinking about it I don’t think it is a good thing

(Participant 3, married, aged 30–45, one child)

We also had women in our study who had experienced IUD insertions as a routine postpartum procedure in the past. All those women described having unpleasant experiences with the IUDs, mainly due to side-effects, and removed their IUDs a short time after. Apart from citing their own experiences with IUDs, some participants also noted the dangers of IUDs, such as complications (even death) due to difficulties in removal, based on the experiences of their family or friends. Still, the IUD was described as if it was a thing of a ‘past time’ and ‘past place’.

### Sub-theme 2b: Placing physical objects inside the body causes damage and injury

Some women, without referring to past experiences of other women or what they heard or read about the historical use of IUDs for married women, expressed fears of placing a foreign object inside the female body. They expressed that doing so may cause pain and damage, and even the thought of putting something inside their body make them uncomfortable. The common reasons given for this were that they felt the IUD might penetrate the uterus wall, cause inflammation, bleeding or might fall out. Some women were fearful of the procedure of inserting the IUD, regarding it as a surgical procedure which was unwarranted.

because for me, I never had any surgical operation done, basically never had a big injury or went under the knife, just like that. That's why I feel that, if you want to put something inside your uterus, you must need to stretch it in or something like that, I just feel like I can’t accept that, especially when it is just for birth control and you don’t have any other illness that requires you to do so. That's why I would not

(Participant 14, not married, 18–30, no children)

### Theme three: It takes two (or one) to decide, depending on the relationship dynamic and contraceptive preferences

This theme captures an important aspect of contraceptive method-use decision-making, which is the decision-making within the dyadic partner relationship. We found that married and unmarried women varied in their opinions of their partners’ involvement in choosing methods. Even within married couples, we found varied attitudes towards their husbands’ involvement, depending on the current method of choice.

### Sub-theme 3a: A husband using a condom is an act of caring

Some of our participants spoke of how their caring, understanding and responsible partners/husbands had enabled them to avoid using female-initiated hormonal contraceptive methods, such as pills or IUDs. There was a sense of acknowledgement and appreciation of their partners’/husbands’ willingness to use condoms. As one woman described:

My partner is also very supportive of this decision to use condoms, and then, he does not ask for much, like he does not ask me to take pills things like that. Since he can live up to using condoms every time [when we have sex], to me, it is a very good thing and it makes me very happy. That's why we always use condoms

(Participant 19, married, aged 18–30, no children)

Another woman also spoke highly of her husband who was very cooperative and always wore a condom or withdrew on time, despite the fact that the majority of men dislike the feeling of a layer of plastic during sex or withdrawing just before reaching orgasm.

that is why I think highly of my husband, he is very considerate towards me, and that is why we can do this, it is not easy at all for a man

(Participant 22, married, 30–45, one child)

In contrast, some other women expressed a sense of frustration and/or distrust towards their husbands’ willingness or consistent/correct use of condoms. One woman cited how her husband does not like the feeling of the condom and insists on using the withdrawal method, which makes her very worried. Another woman, while acknowledging that condom is an effective method which they are currently using, mentioned that her husband sometimes is not willing to wear one during sex.

One woman (married, aged 30–45, one child) who underwent termination of an unintended pregnany and had IUD inserted described how she felt ‘in control’ by not having to rely on her husband using the condom. She noted she did not involve her husband in choosing a female-initiated contraceptive method process, because she believed that ‘things like this, women should make their own decisions’. As she put it:

with IUDs, I am the one who is taking control, after having the IUD I feel this way, see, with condoms, in fact the control power is with the other half, see, am I right, you can’t do much if they don’t use condoms

(Participant 4, married, 30–45, one child)

The above woman was one of three married women in this study who were currently using IUDs as their contraceptive methods. A second woman, who had three children, did not involve her husband in choosing a female-initiated contraceptive method as well because, as she put it, ‘not many men do a serious research on this matter’. The accounts of these two women capture a sense of frustration that, unlike themselves, their husbands do not take contraception ‘seriously’.

### Sub-theme 3b: Young women should be responsible for taking care of their body

The decision-making dynamic took a slightly different perspective for our participants who were unmarried and not living together with a partner. Majority of those unmarried women also preferred the use of condoms by their partners, mainly because they are not having sexual intercourse frequently. Therefore, for them, it was not worthwhile to take pills and suffer from side-effects. However, the decision to use condoms was primarily insisted upon by the women themselves, with their partners being expected to comply. They affirmed that ensuring a method of their preferences was an act of ‘being responsible for self’. As one participant put it, ‘he has no right to decide’. Another participant also described how she makes all the decisions about contraception and her partner does not interfere nor care. Similarly, one participant expressed that despite her partner preferring not to use the condom, for her, taking pills instead was not an option because she did not like the side-effects that came with them.

he always wants me to be on the pill. Yeah. ‘Cause – yeah, of course, it feels better, but – no, I don’t wanna feel moody and I don’t wanna look ugly again with all this pimple

(Participant 11, not married, aged 18–30, no children)

There was also a consideration for sexually transmitted infection (STI) prevention by using condoms among unmarried women.

To me, it is not only about the condom being able to prevent unplanned pregnancy, it is also about it being able to prevent STIs. Although I am willing to believe that my partner is clean, but I think, to me, it is very important [preventing STIs]

(Participant 1, not married, aged 18–30, no children)

Therefore, young unmarried Chinese women often saw requesting condom use as an act of self-protection against unintended pregnancies, STIs and potential side-effects of other female-initiated methods. In their relationship dynamics, they were highly motivated to use condoms and it was implied that their partners were expected to conform to their preferences.

### Theme four: It is not necessary to seek medical advice in choosing contraceptive methods

When asked if they had ever seen a doctor to discuss contraception or considered seeing one, some participants felt confused by the question as to why a doctor would be needed. For example:

I have not, yet, I never thought about that [seeing a doctor], because I felt that I can resolve this problem myself, but I don’t understand, seeing a doctor, do you mean seeing a doctor and doctor give me some advice or something? I probably would not, I would probably just decide on my own

(Participant 14, not married, aged 18–30, no children)

### Sub-theme 4a: Did not need to consult a doctor regarding contraceptive methods

Some women cited cultural and national context differences between their home country and Australia as reasons why they thought seeing a doctor was unnecessary. They described that people did not have to go to a doctor specifically for asking which contraceptive method to choose. Women’s accounts also implied that even for IUD insertions requiring a health professional, doctors’s involvement was usually procedural. One woman, who had experienced an IUD insertion in China postnatally described:

no one would consult a doctor about how to avoid pregnancy/birth control, [doctors] normally and only say, have you given birth yet, if you have, let's put the ring in, they all say things like this, then put the ring in. They would not discuss too much, no one would tell you if there is any effect of the ring or if it hurt…. In China, the doctor-patient relationship, sometimes it is very tense, and sometimes, sometimes there is not much talk, because they fear, fear if they say something and it causes some reaction, then the patient would come to the hospital to dispute

(Participant 22, married, aged 30–45, one child)

Regulations around prescribing contraceptives also added to the contextual differences between the two countries. Participants mentioned that they did not need a prescription for contraceptive pills or devices in China. These could be purchased at a pharmacy. One woman in our study also mentioned that she bought contraceptive pills when she was back in China because it was cheaper there.

### Sub-theme 4b: Needing to use contraception is not an illness to be treated

Also common among our participants was the view that one only needed to see a doctor to treat illnesses or fix something, e.g., when someone is accidentally pregnant and needed an abortion or they were suffering from complications or having gynaecological problems.

I think you only need to see a doctor when you have a problem that you need to fix, like the problem is already there, like falling pregnant accidentally. I don’t think I would choose to go and specifically see a doctor and ask how to avoid falling pregnant…. also, I don’t know if it is the same in other cultures or countries, but in China, people tend to conceal one’s sickness rather than to tell the doctor, sometimes you would have a cold but you would not want to go see a doctor. First, it is the trouble, second, after seeing a doctor, you would feel that your body is in an inferior state, you would have that sort of psychological hint

(Participant 9, not married, 18–30, no children)

Some women cited that they did not feel the need to consult a doctor because the condom as a contraceptive method was common knowledge and there was no need to consult a doctor. When specifically probed about whether they ever felt any difficulties in choosing contraceptive methods, many participants, especially those who preferred condoms, expressed that it was a straightforward thing to think of using condoms, therefore they had no difficulties.

### Sub-theme 4c: Can get contraception related information elsewhere: reliance on Chinese language websites, social networking forums and WeChat groups

One of the other reasons given by participants for not needing to consult a doctor was that they could easily find information elsewhere. Many participants expressed their preferences for Chinese language online resources, because they were easier to read and comprehend. Zhihu, which is a Chinese equivalent of question-and-answer website Quora, was cited by several participants as a reliable and relatively authentic source for contraception related information. Also among the mentioned Chinese websites were Guoke and DingXiangYuan, which are China-based popular science news and knowledge websites. Apart from those websites, there was a general inclination for hearing other women’s real-life experiences with the contraceptives by reading reviews or asking questions on social networking and discussion boards, Weibo (the Chinese equivalent of Twitter) and Wechat groups.

One particular woman, who had chosen a hormonal IUD after it was recommended by her general practitioner, decided to participate in our study to tell her story. She stressed that hormonal IUD was not as bad as some people wrote online. She recounted how she was scared of the side-effects of the hormonal IUD after reading many negative reviews online. However, after giving it a try, she was satisfied with her chosen method.

‘I wanted to be in this study, because I did not want it to end up with all the people saying bad things about it [hormonal IUD]. In fact there are people who used and liked it, in fact sometimes people do not bother to say something, because usually there is not a problem, you don’t pay attention to it, you are busy with other things. Those who talk about those things all day are those who had unresolved problems, that is why we hear unhappy views everywhere

(Participant 4, married, aged 30–45, one child)

## Discussion

This interview-based qualitative study explored Chinese migrant women’s perceptions of, preferences for and experiences with choosing a contraception method in Australia. We identified four main themes from our participants’ accounts of the contraceptive methods that they knew or used and the processes they took to choose contraceptive methods either in Australia or China. Based on the principles of critical realism, we will now explore how those perceptions and experiences of women are likely to be shaped and generated by personal, cultural and social contexts and mechanisms.

Our findings with respect to women’s perceptions and views of contraceptive methods suggest that there is a strong sense of rejection of hormonal contraceptive methods due to fear of side-effect and the profound belief of ‘every medicine is part poison’. The most commonly feared side-effect was reduced or absent menses as a result of hormonal contraceptive use. Such finding is in line with earlier studies looking into ethnic Chinese women’s beliefs and experiences with contraceptives in other Western countries [29, 30]. The women’s responses signalled an underlying belief system related to being a woman, menstruation, fertility, aging and body constitution. Body constitution is a key indicator of a person’s physical wellbeing in Traditional Chinese Medicine (TCM) [31], which is deeply rooted in the Chinese view of health and practices [32]. There are nine types of constitutions, including balanced and peaceful (ideal), Qi (vital energy)-deficiency, Yang (positive force)-deficiency, Yin (negative force)-deficiency, Phlegm-dampness, Damp-heat, Stagnant Blood, Stagnant Qi (vital energy), and Inherited Special Constitutions [31]. In TCM, when the body’s constitution is other than balanced and peaceful, it would mean that there is disharmony within the body which could be identified by associated symptoms) [31]. As for women, an unbalanced constitution could often manifest itself in the form of menstrual disorders, as the classic saying goes ‘in women, blood is the ruling aspect, with blood and Qi being interdependent’ [33]. Menstruation once-a-month is regarded as a process where old blood is cleansed out of body and new blood begins to generate inside the body [33–35]. Regular, monthly menstruation is seen as a signal of fertility as well as general health and the absence of menses was viewed as the most serious menstrual crisis [33–35]. Therefore, the most prominent menstrual therapy in TCM is based on the notion of ‘replenishing and vivifying’ female blood by invoking regular menstrual blood loss [33–35]. Such concepts around body constitution and menstruation could potentially helpful to explain why some women in our study were fearful of hormonal contraceptives that might reduce/eliminate the amount of menstrual bleeding. An earlier 5-year follow up study on the safety of levonorgestrel-IUD among Chinese patients also found high discontinuation rates among users, mainly due to amenorrhea and irregular bleeding [36]. Another study also found that worry about future fertility was the most common anxiety in relation to IUDs and implants among post-abortion Chinese women [37].

As for IUDs, the historical and societal context of IUD use for parous women in the past and experiences of other family members, friends and themselves made this a non-option for women who had other choices or who are not married or do not have children yet. In China, it was reported that in 2016, IUDs accounted for 53% of all contraceptive methods that were used by married women of childbearing age [38]. However, there is a trend of decline in the use of IUDs, especially in economically developed cities/provinces [38]. For example, in Beijing, condoms were the main method of birth control in 2016, with a 78.9% prevalence rate, and the IUD prevalence rate was just under 20% [38]. Condoms were also found to be the most prevalent contraceptive method (61.4%) among unmarried women in China, followed by rhythm (19.8%) and withdrawal (25.4%) methods [39]. There are no statistics of the prevalence of contraceptive method use among Chinese women living in Australia. However, accounts of women who were interviewed in this study suggest that for them, the IUD is not a desirable option that they would consider at first. There was also concern about a foreign object being placed in their body which invoked fears about both the procedure itself and the potential complications. Such concerns, however, are not unique to Chinese women living in Australia. Worries about infection, perforation, expulsion as well as troublesome bleeding have been identified by several studies amongst different population groups [40–43].

Given that hormonal contraceptives and non-hormonal IUDs are generally not preferred by women, at least initially, we found that women usually intend/prefer to use condoms with or without withdrawal and fertility-awarenss based methods. Such intentions can subject to influences from multiple factors, including partner preferences and provider recommendations [44]. In our study, partner preferences were found to be more influential among married women than unmarried and non-cohabiting women. There was a sense of appreciation among married women that their choice of the condom as a contraceptive method was granted and fulfilled by their husbands. A similar study from mainland China also revealed how women often expressed that their husband made the ‘caring’ decision to use condoms as a contraceptive method [45]. A study conducted by Miller and Pasta found that, among married couples who were currently using condoms or diaphragms, husbands were likely to conform to their wife’s contraceptive method desires rather than their own [46]. On the other hand, when women were not confident in their husbands’ use of condoms, they are more likely to intend to change methods [46]. Also, when method choice was female-controlled, women tended to have more influence on the decisions than those required male cooperation [46, 47]. This was also the case for some women in our study who chose IUDs without involving their husband in the decision-making process. By contrast, for unmarried women, we found that they placed little to no weight on the preferences of their partners.

We found that for both the married and unmarried Chinese migrant women, influence from providers was mostly absent from their decision-making processes. Our findings revealed an underlying belief that one only need to see a doctor to treat illness, and that the need for contraception was not illness. A prior study conducted among Chinese immigrants also found that Chinese immigrants believed the doctor’s role was to prescribe medications [48]. They tended to seek medical advice only when their conditions were perceived as serious [48]. It was found that for perceived minor illness, Chinese migrants preferred self-management [48, 49]. In Australia, on study found that around every six out of 100 general practice consultations are contraceptive management related [50]. Apart from the prescription of medications, general counselling and education were one of the most common types of non-pharmacological management of contraception problems [50]. Unsurprisingly, it also reported that women who speak a language other than English were less likely to have contraceptive management related encounters with general practitioners compared to English-speaking only women [50].

Our study results show that women often acquired their information about contraception on Chinese language websites and platforms, because of the ease of reading. Majority of the participants mentioned or were aware of the Australian family planning specific websites, pointing to system and language barriers as well as inequalities in access to health information and services. We also found that women preferred experiential information on contraceptive methods by learning about real-life experiences of other women. This finding is similar to other studies where women expressed seeking experiential information on contraceptive methods [51, 52]. In most cases, healthcare providers or local Australian contraception related evidence-based resources were usually not the first point of contact for information and advice. This finding should have important policy and practice implications for delivering reproductive health services to Chinese migrant women in Australia, specifically in terms of contraceptive services.

## Implications for policy and practice

This study has several implications for policy and practice. It is undoubtedly challenging to deliver the current best practices in contraception counselling, such as patient centred care and shared decision-making [53], to Chinese migrant women who are making their contraception decisions without involving healthcare providers. However, when women do present to healthcare providers with contraception related problems, in line with best practices and on the basis of respecting women’s reproductive autonomy and rights, proactively eliciting and acknowledging women’s preferences and concerns around contraceptive methods is likely help women make informed decisions. For example, it may be necessary to provide explanation to how contraceptives work, especially hormonal ones, and how they impact menstrual bleeding patterns and subsequent fertility, as these were among the major concerns of women. Where necessary, explaining the differences between current day copper and hormonal IUDs and those IUDs used in the past, notably SSRs, is likely to help addressing women’s concerns around IUDs. It might be also imperative for healthcare providers to explain the procedures involved in IUD insertions, especially around pain and potential complications. In all cases, providing evidence-based information on the side-effects and their probabilities is likely to ensure a method choice that is concordant with women’s preferences and based on evidence-based information.

Most importantly, to better cater to women’s information, communication and support needs in terms of contraceptive method-choice decision, interventions may need to expand their focus from the counselling domain to broader health literacy, health education and promotion domains. Such interventions can serve to provide women equal access to evidence-based and Australian-country specific contraceptive related resources. Interventional strategies could include developing culturally and linguistically sensitive contraception related educational materials, decision support tools and interactive workshops/seminars and delivering them through channels that are easily accessed by Chinese migrant women. One of the implications from our study is also the importance of integrating user testimonies into the health education and decision support materials. Also important is improving migrant women’s awareness of healthcare providers’ role in providing contraceptive related counselling and related services. It is often reported that immigrants’ health seeking behaviours are influenced by their prior experiences of accessing healthcare in in their country of origin [12, 54]. Therefore, integrating messages, such as availability of counselling and other services, to health education materials, welcome to country/university packs is likely to help improve health system awareness.

## Limitations

Despite our efforts to recruit women who are seeking contraception services at family planning clinics, we were only able to recruit one participant from such venues due to low representation at such clinical settings. Therefore, the views and perceptions expressed by women in our study may not be representative of those women who actively sought medical help for managing their contraceptive needs. We also only recruited women who had been living in Australia for no more than 10 years to minimise the impact of acculturation and to target those who are most likely to be culturally and linguistically distinct. Also, due to voluntary nature of recruitment, those women who had decided to participate in our study might be more open to discussing sexual and reproductive matters and more confident with their current chosen contraceptive methods than those that who did not participate. Therefore, our study results should be interepreted in light of the participants’ characteristics (Table 1).

## Conclusion

Our findings suggest that Chinese migrant women’s perceptions and experiences of contraceptive methods are influenced by complex personal, cultural, societal and inter-relational factors. The findings are likely to be useful in increasing healthcare professionals’ and policy makers’ understanding of Chinese women’s contraceptive method preferences, beliefs and behaviours and improving responsiveness/sensitivity of health services and interventions to cater to women’s decision-making needs.

## Supplementary Information

**Additional file 1:** Interview script and topic guide.

## Data Availability

The data generated during the current study will be available from the corresponding author on reasonable request and in accordance with the consent and ethical approval.

## Abbreviations

IUD: Intrauterine device
SSR: Stainless steel ring
STI: Sexually transmitted infection
TCM: Traditional Chinese Medicine
