# Supplementary material for: ‘Every medicine is part poison’: a qualitative inquiry into the perceptions and experiences of choosing contraceptive methods of migrant Chinese women living in Australia
Source: BMC Womens Health. 2021 Mar 8;21:100. doi: 10.1186/s12905-021-01226-3 (PMC7938530; doi:10.1186/s12905-021-01226-3)
Supplement: Supplementary file 1 — Additional file 1: Interview script and topic guide. [file 12905_2021_1226_MOESM1_ESM.docx]

**Contraceptive Choice Decisional Needs Assessment of Chinese Women Living in Australia**

**Telephone and face-to-face interview script**

- Introduce self
- Confirm that the participant has received the participant information statement, participant consent form and copy of decision aid tool.
- [if not already been returned] read out the consent form and obtain verbal consent
- [if already been returned] verbally confirm consent
- Conduct the interview in accordance to interview topic guide

[*for telephone interview*] Hi, this is Hankiz from the University of Sydney, Can I speak with _______________please?

My name is Hankiz and I am a current PhD student at the University of Sydney. You have previously expressed an interest in a study that I am currently conducting about contraceptive choice decisional needs assessment of Chinese women living in Australia. Is that right?

Great. Can I please confirm that you have received the participant information statement, consent form and decision aid tool that I have previously sent you via _______?

Great. As stated in the participant information statement, being in this study is completely voluntary and you can withdraw at any time without affecting your relationship with any researchers involved in this study or your healthcare provider. This study has been approved by the Sydney University Human Ethics Committee and all information you give as part of this study will be strictly confidential. After reading the participant information statement, do you have any questions or concerns that you would like to ask?

[*address the questions*]

[if not already been returned] If you are willing to be involved in the study, I will read out the consent form for you. You can stop me if you have any questions. [Hankiz reads out the consent form]

[if already been returned] I have already received your consent form via__________. Before proceeding with the interview, can I confirm with you that you consent for this interview to be audio-recorded?

Interview begins [refer to interview topic guide]

**Interview topic guide for Chinese women participant [Part One]**

(based on and adapted from Jacobsen, M. J., et al. (1999, 2013). "Decisional needs assessment in populations.”)

**Beginning**

- Thank you for your time and for agreeing to being interviewed.
- This interview is about learning more about your information, communication and support needs when choosing a contraceptive method.
- Everything you say is strictly confidential.
- The interview will last for about an hour.
- You can choose not to answer questions that you do not want to. If you wish to not to be interviewed anymore, you can do so at any time.

Do you have any questions before we begin?

**Opening Questions**

1. Just to start with, could you tell me how long have you been in Australia?
2. How do you describe your overall experience in accessing and receiving healthcare in Australia?
3. Were there major health decisions that you had to make since you were in Australia?

*Probe:*

- such as a treatment decision for a medical problem, or lifestyle decision?

*if no major health decisions were made, probe:*

- *or you can tell me about any circumstances where you had to visit a healthcare professional.*

1. How was your experience in making above mentioned decision/s (or when you visit your doctor)?

*Probe:*

*Did you have to go to a doctor (if not mentioned before)*

- *How do you describe your involvement in making the medical decisions?*
- *How do you describe your doctor’s role in making the decisions with you? Why?*
- *(How do you describe your role during consultation? Why?*
- *How comfortable did you feel about asking questions from the doctor?*
- *Can you tell me the reasons why you did not go to the doctor?*
- *Was there anyone else involved in making this decision with you? What was their role? What was your preference for their involvement?*

1. Now, let us move on to the focus of this study, contraception decisions. Is contraception something you think about in relations to your health? Why?
2. How important is contraception decision to you compared the other health decisions that you have to or had to make?
3. Have you ever used or are you currently using any types of contraception?

*Probe:*

- *What types of contraception have you used or you are currently using?*

**Knowledge and beliefs**

1. What do you see as the main advantages/benefits of the contraception method/s you have used? (note: repeat the question for each of the methods that were given if there were more than one)
2. What do you see as the main disadvantages/risks of the contraception method/s you have used? (note: repeat the question for each of the methods that were given if there were more than one)
3. What are the other contraception options that you are aware of?
4. What do you see as the main advantages/benefits of the other option? (note: repeat the question for each of the options that were given)
5. What do you see as the main disadvantages /risks of the other option? (note: repeat the question for each of the options that were given)

**Values**

1. Among the above-mentioned advantages/benefits of contraception methods, which are most important to you when thinking about choosing a contraception method?
2. Among the above-mentioned disadvantages/risks of contraception methods and options, which are most important to you to avoid when thinking about choosing a contraception methods?
3. Do you have any preference for any particular types of contraception? Why?

**Decisional Conflict**

1. Let’s talk about the difficulty with making decisions about contraception. How do/did you feel when you think about making a decision about contraception?

*Probe*

- *How do/did you feel about the importance of making this decision?*
- *How do/did you feel about your confidence in knowing what to do?*
- *How do/did you feel about your confidence in making the right decision?*
- *How do/did you feel when faced with more than one options available?*
- *How did you feel about the timing of the decision?*
- *Are/were there any negative feelings when thinking about contraception, such as worry, anxiety, pressure or shame?*

1. What things do you think make/ made the decision difficult for you? Why?

*Probe:*

- *Are/were there any difficulties in getting information about contraception options, benefits and risks? Why? Why not?*
- *Are/ were there difficulties on getting information on chances of benefits and harms? Why? Why not?*
- *Are/were there confusion about the information you have? Why? Why not?*
- *Have you felt any pressure from others when making decisions about contraception?*
- *How did you feel about your personal skills in helping you making the decision? Why?*

**Support and resources**

1. What would be the steps for you in making contraception decisions?

- What types of information do you look for?

1. Who or which places do you usually go to for support in making contraception decisions? Why?
2. Do you have any preference for any particular type of healthcare providers in assisting your contraceptive decisions?

Probe:

- Such as gender, language and specialty? Why?

1. Have you ever been to a healthcare professional to discuss contraception?
2. How comfortable were/are you talking to your healthcare provider about contraception?
3. (if any) Based on your previous visits to the healthcare professional to discuss contraception, could you tell me what things did the health professional did during the consultation?

*Probe*

- *What kind of information did she or he give to you?*
- *Did your healthcare provider explain the benefits and side effects of an option/ options to you?*
- *Did you ask any questions? What questions?*
- *Did you have chance to deliberate your thinking on each option and your personal preference?*
- *How satisfied were you with your experience? Why? Why not?*

1. (if any) how do you describe the healthcare providers’ role in making contraception decision with you?
2. (if any) how do you describe your role in making contraception decision during consultation with your healthcare provider?
3. what do you think should be your doctor’s role in terms of assisting you in making the contraception decisions?

- What kind of information would you expect from your provider?
- What kind of support would you expect from your provider?

1. Who else is usually involved in making contraception decision for you?
2. What is their usual role in making this decision (i.e. the person mentioned above, repeat the question with each person)
3. What would you prefer their role to be in making this decision with you? (i.e. the person mentioned above, repeat the question with each person)
4. Overall, what do you think would help you in making contraception decisions?
5. What will hinder you (get in the way of) making the contraception decision?
6. Is there anything else that would help you overcome these barriers to decision making?
